# A Seamless Hybrid Phase II/III Design With Bayesian Interim Subgroup Selection

**DOI:** 10.1002/sim.70144

**Published:** 2025-06-03

**Authors:** Benjamin Duputel, Nigel Stallard, François Montestruc, Sarah Zohar, Moreno Ursino

**Affiliations:** ^1^ Inserm, UMRS 1346, Université Paris Cité, Inria HeKA Paris France; ^2^ eXYSTAT Malakoff France; ^3^ Warwick Clinical Trials Unit, Warwick Medical School University of Warwick Coventry UK

**Keywords:** binary, horseshoe prior, log‐rank test, survival analysis

## Abstract

Population selection is a crucial subject in clinical development nowadays as personalized medicine is growing in interest. Evolution in biomarker scanning techniques allows for the composition and detection of sub‐populations of interest when analyzing new drug responses in a disease. Seamless adaptive trials could allow for subgroup analysis with the selection of the most promising population at interim analysis. We propose a hybrid Bayesian design for seamless Phase II/III trials with binary and time‐to‐event outcomes for the first and second phases, respectively. In this work, at interim analysis, several prior distributions, including shrinkage prior, are compared to possibly select/discard a population, and a final test using a conditional error function as a combination method testing procedure to control the frequentist type I error is used. Simulation studies showed that the logistic regression model performs better than frequentist testing for the population selection problem when the subgroup should be selected. Shrinkage prior distributions tend to be more conservative than simpler normal distributions as studies that would have ended positively are stopped at interim analysis.

## 1 Introduction

For the last decades, interest in flexible designs for clinical trials has grown significantly. Bothwell et al. [1] reported in their review paper that almost no adaptive clinical trials were found in the 1980–1990's whereas since 2010 more than twenty a year are recorded. Adaptive designs (AD) are appealing because they meet ethical standards by offering faster methods to identify efficient treatments for patients and discontinuing ineffective ones. They can be more powerful than classical studies (by sample size re‐estimation for example) or use a smaller sample size (seamless trials and combination testing) while controlling error rates. They also offer logistical and economical benefits. The counterpart is that these methods need more upfront planning and modeling.

Usually AD are constructed around interim analyses (IA) that lead to a decision of whether or not to apply a predetermined adaptation. The most common adaptation is the group sequential design (GSD) as proposed, for example, by Pocock [2], in which repeated analysis of an outcome enables early termination of the study once a significance threshold is reached. The idea has been extended in the books by Jennison and Turnbull [3] or Whitehead [4] o allow early stopping of the trial if efficacy analyses reach a futility threshold. Other adaptations consider sample size reassessment [5], dropping ineffective treatment or dose arms [6, 7], or selection of previously identified population [8].

For population selection problems, trials can be constructed as seamless studies (two phases combined in a unique protocol), where the Phase II part, that aims at selecting the population that could benefit from the therapy, is combined with the confirmatory Phase III part. Pooling both phases together has the benefit of reducing the global sample size while controlling type one error rate. Seamless Phase II/III trials with population selection [9] has been studied in a few papers. For instance, Jenkins et al. [10] or Spiessens and Debois [11] proposed combination test methods to address the issue. Those methods select the population on the base of interim responses, and use combined values of statistical tests from both stages at final analysis. Friede et al. [12] proposed a two‐stage conditional error procedure. Similar to previous methods, test statistics obtained at an interim analysis at the end of the first stage are used to select a population, with a final conclusion based on data from both stages. Overall type I error rate control is achieved by considering the correlation between normally distributed test statistics from the two stages and in different sub‐populations. This correlation structure has been studied and extended to multi populations, multi arms trials by Chen et al. [13]. Miao et al. [14] recently proposed a subgroup analysis using progression free survival and overall survival in a combination test for their seamless Phase II/III trial. Bayesian theoretic designs have also been proposed as in Brannath et al. [15], Kimani et al. [16], or more recently, Ballarini et al. [17]. These methods use maximization of utility functions (responding to a specific need as safety and efficacy, or logistical costs, etc.) to decide if the trial should proceed to the second stage with the subgroup or the full population.

In this paper, we propose a two‐stage design with an interim sub‐population selection step, employing a selection step based on the posterior distribution of parameters from a Bayesian logistic regression, rather than a utility function. A binary (dichotomised) survival rate endpoint is assumed for the first stage of the study and overall survival for the final analysis. Overall survival is defined as the time between inclusion and death by any cause; a patient is considered censored if he is either lost to follow‐up or alive at the time of analysis. To account for the selection step and try to avoid multiplicity issues, we adapted the conditional error function approach of Friede et al. [18]. Comparison between multiple prior distributions is made, and a frequentist two‐stage design based on a statistical test for selection is also used for comparison. The remainder of the manuscript is structured as follows. In the next section, we briefly present the real clinical trial Atalante‐1 that serves as a motivational case study for this research. In Section 3, the model and design are described and illustrated. In Section 4 the parameters and the scenarios of simulation studies are presented along with the results for two distinct prevalence rates of the subgroup, as well as for another selection threshold for the Bayesian designs. Finally, we discuss the benefits and limitations of the proposed method in Section 6.

## 2 Motivating Study

The Atalante‐1 clinical trial (NCT02654587) used a seamless Phase II/III design with a Fleming single arm method [19] for the first stage and a stratified log‐rank test for the second one. The study compared the experimental treatment (Tedopi) to the best standard of care (Docetaxel or Pemetrexed) in terms of survival rate at 1 year (stage 1) and overall survival (stage 2) on non‐small‐cell lung cancer. The operating characteristics of this trial included a type I error rate of 2.5% and a power of 80%. The sample size required was 84 evaluable patients for the first stage of the trial, under the null hypothesis of a 12‐month overall survival rate of 25% in the treatment group, and an alternative hypothesis of 40%. At the interim analysis, the study could either stop for futility or proceed to Phase III to compare overall survival with the control using a frequentist approach. For the second phase, aiming for a power of 80% to detect a significant difference with a two‐sided significance level of 5%, 363 new patients were planned to be evaluated. This was under a 2:1 randomization scheme (in favor of the experimental arm) to detect a Hazard Ratio (HR) of 0.7, assuming a median survival of 7 months in the control arm, which would imply a 10‐month median survival in the treatment arm. At the time of the planned interim analysis when the first 103 patients reached 12 months of follow‐up, the decision was taken by the sponsor to prematurely stop the accrual due to the coronavirus disease 2019 (COVID‐19) pandemic, which was rapidly expanding with a strong concern about its impact on patient safety and data integrity. Thereafter, treatment and follow‐up continued for the ongoing 219 patients already randomized. Due to this early accrual discontinuation, the data were unblinded and analyzed for the first 103 patients. A subgroup of interest from a stratification factor was identified based on a clinical and biological rationale [20]. At the time of final analysis, this subgroup was analyzed in the overall population of 219 patients as a post hoc analysis. A confirmatory study (Artemia) is ongoing to confirm the treatment effect in this population of interest. Motivated by this experience, our research focused on exploring new Bayesian methods for seamless design where a subgroup is pre‐identified at the design stage and prospectively selected or discarded during the trial.

## 3 Model and Design

**FIGURE 1:**
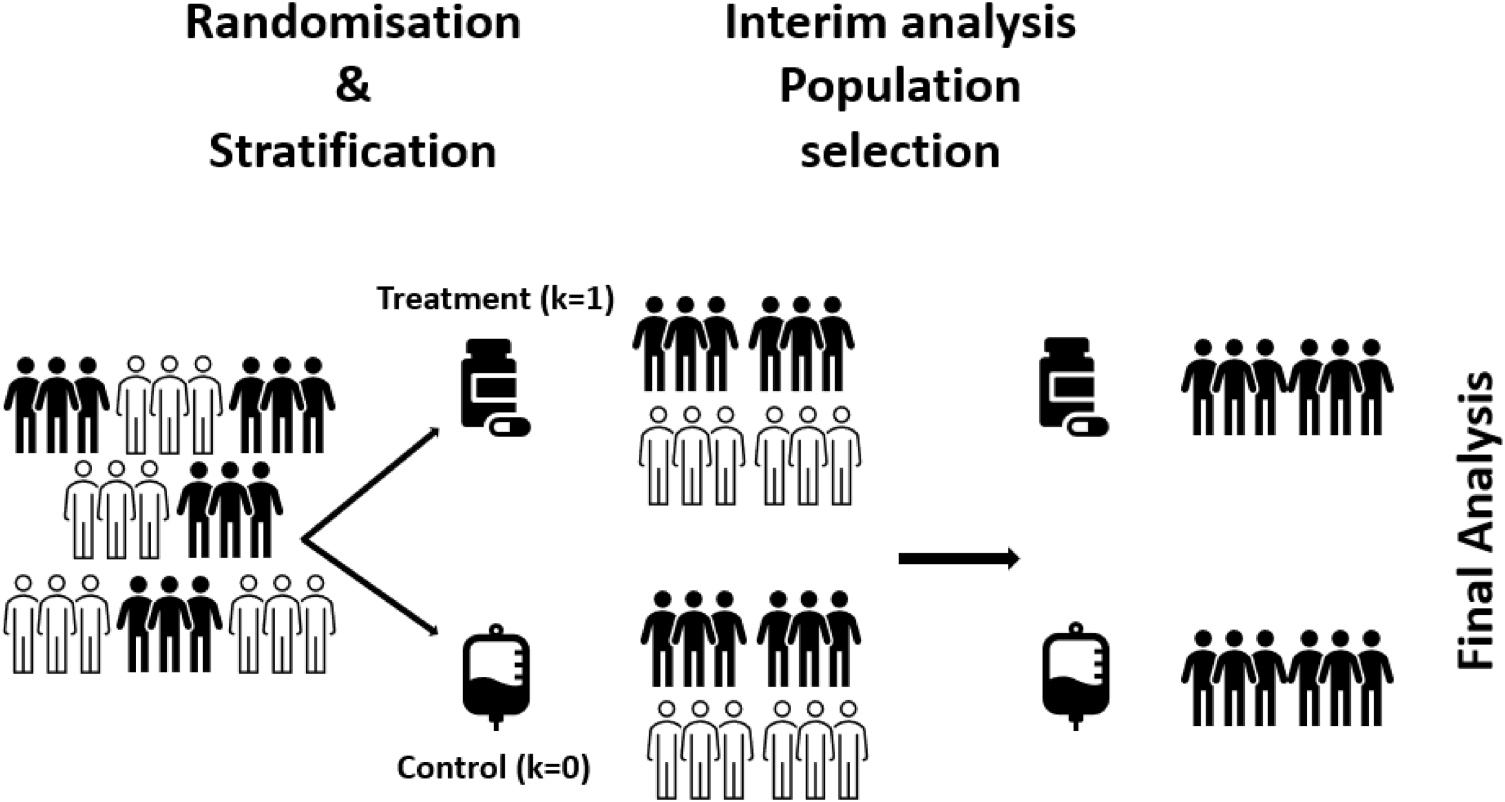
Trial scheme with selection of the relevant population at interim analysis based on binary survival rate endpoint. Then, the final analysis is based on a time‐to‐event endpoint. In this example, the sub‐population is selected at interim, and the treatment is found superior to the control at final analysis.

Our method uses the same endpoints as the case‐study trial. The mortality rate at 1 year is used for decision in the first step of the seamless trial (Phase II), and overall survival is considered for the final analysis (Phase III). At the interim analysis, a Bayesian approach, as described in the following subsections, is used as a selection tool for the determination of the most promising population (possibly both) for the second step of the study. In the Phase III of the trial, we use a frequentist testing approach that accounts for the first stage data via a conditional error function for the final efficacy analysis. In addition, a closed testing procedure is considered when F and S populations are simultaneously analyzed at the end of the trial. The overall trial design is illustrated in Figure 1. The proposed design adopts a Bayesian approach for subgroup selection at the interim analysis; however, through the application of the conditional error function and the closed testing procedure, it guarantees a *p*‐value adjustment in the final analysis, reducing the inflation of the frequentist type I error rate.

### 3.1 Notation

Let k=0 and k=1 be the indicator of control and treatment group respectively. In this work, we consider only two arms with a 1:1 randomization scheme. Let $N_{\mathrm{II}}$ and $N_{\mathrm{III}}$ be the fixed sample size of the Phase II and Phase III parts of the trial in each arm. Let the full population be denoted by F, and the subgroup of interest be denoted by S (and its complementary $S^{C}$, $F=S\cup S^{C}$). Let $t_{i,k}$ and $c_{i,k}$ be the time to event (death in our motivating example) and time of censoring for ith patient in treatment group k. Let $y_{i,k}$ be the first occurrence between event and censor (lost to follow up or alive at the time of analysis) $y_{i,k}=\min\left(t_{i,k},c_{i,k}\right)$ with $t_{i,k},c_{i,k}>0$, and let $\nu_{i,k}$ be the event indicator meaning that $\nu_{i,k}=1$ if $\;y_{i,k}=t_{i,k}$ or $\nu_{i,k}=0$ if $y_{i,k}=c_{i,k}$. Let $D_{k}^{p}$ denote the data from group k at phase p=II or III, $D_{k}^{p}=\left\{N_{k},y_{k},\nu_{k}\right\}$, where $N_{k}$ is the sample size of the group k at time of data collection (i.e., $N_{k}=N_{\mathrm{II}}$ or $N_{k}=N_{\mathrm{III}}$), and, $\nu_{k}$ and $y_{k}$ are vectors of length $N_{k}$ containing all values of $y_{i,k}$ and $\nu_{i,k}$.

### 3.2 Stage 1‐Phase II

A dichotomous endpoint, survival indicator at some specified time, $t^{*}$ (e.g., 1 year as in the motivating example), is used for the interim analysis decision. A patient can either be dead or alive at $t^{*}$, patients censored before the observation of their status at $t^{*}$ are excluded from the survival rate comparison. Interim analysis occurs when $N_{\mathrm{II}}$ patients have been recruited in both groups and have completed the follow‐up period. We denote by $y_{i,k}^{*}$ the observation at time $t^{*}$, where

$$y_{i,k}^{*}=\left\{\begin{array}{l} 1\;\text{if}\;y_{i,k}\geq t^{*}\\ 0\;\text{if}\;y_{i,k}<t^{*}. \end{array}\right.$$

Let $p_{i,k}\in \left[0,1\right]$ represent the probability of being alive at time $t^{*}$ for ith patient in the kth group, then we have $y_{i,k}^{*}∼\text{Bernoulli}\;\left(p_{i,k}\right)$. We assume that $p_{i,k}$ depends on the sub‐population and on the group k. We use the logit link function, as recommended by Albert and Hu [21], to link $p_{i,k}$ to patient's covariates, that is $\;\text{logit}\;\left(p_{i,k}\right)=\theta^{T}X_{i,k},$ with $\theta =\left(\theta_{0},\theta_{S},\theta_{T},\theta_{\mathrm{TS}}\right)$ representing the parameter vector and $X_{i,k}$ as the covariate indicator matrix that contains information on the patient's group (either F or S and belonging to either the control or treatment arm). Regarding the parameter vector: $\theta_{0}$ is the intercept and represents the effect of the control on the full population; $\theta_{S}$ indicates the shift from $\theta_{0}$ for the global effect across both control and treatment groups within the sub‐population; $\theta_{T}$ represents the shift of the treatment effect in the full population relative to the control; lastly, $\theta_{\mathrm{TS}}$ is the interaction term between the treatment arm and the sub‐population. Prior distributions on these parameters are introduced in Section 3.5.

### 3.3 Interim Selection Rules

At the end of the Phase II part, the promising population (possibly both) is selected for the second stage of the study. After conducting the Bayesian analysis, posterior distributions are utilized to determine whether the trial will continue with F, S, or be declared futile. If the treatment demonstrates efficacy in both S and F, the trial would continue with the full population. However, the final analysis would take both populations into account. In this scenario, while the treatment benefits the entire (full) population, the treatment effect may notably stronger in the sub‐population. The interim analysis focuses on $\theta_{T}$ and $\theta_{\mathrm{TS}}$ as the parameters of interest. This means that the decision to either continue or stop the study will be based solely on these two parameters, under the expectation that the efficacy of the control has already been established in the global population. We therefore propose that, as summarized in Table 1, the first step in the interim analysis decision‐making process involves examining the posterior distribution of $\theta_{\mathrm{TS}}$ and its probability of being greater than a predetermined threshold ($\zeta_{e}$), specifically focusing on whether it exceeds an interim limit threshold ($\tau_{S}$), $\mathbb{P}\left(\theta_{\mathrm{TS}}>\zeta_{e}|D_{k}\right)>\tau_{S}$. The analysis of the $\theta_{T}$ parameter follows the same rule, using $\tau_{F}$ as the threshold. If $\mathbb{P}\left(\theta_{\mathrm{TS}}>\zeta_{e}|D_{k}\right)>\tau_{S}$ and $\mathbb{P}\left(\theta_{T}>\zeta_{e}|D_{k}\right)>\tau_{F}$, then the trial proceeds with an analysis in both S and F (S&F). If $\mathbb{P}\left(\theta_{T}>\zeta_{e}|D_{k}\right)<\tau_{F}$, but if $\mathbb{P}\left(\theta_{T}+\theta_{\mathrm{TS}}>\zeta_{e}|D_{k}\right)>\tau_{1}$, where $\tau_{1}$ is another interim limit threshold, then the trial continues in the subgroup as a treatment effect is found in the subgroup, otherwise the trial will be halted for futility. If $\mathbb{P}\left(\theta_{\mathrm{TS}}>\zeta_{e}|D_{k}\right)<\tau_{S}$, the next steps depend on the analysis of $\theta_{T}$: if $\mathbb{P}\left(\theta_{T}>\zeta_{e}|D_{k}\right)>\tau_{F}$, then the trial continues with a focus on F only; otherwise, if $\mathbb{P}\left(\theta_{T}>\zeta_{e}|D_{k}\right)<\tau_{F}$, the trial is stopped for futility. For simplicity, $\zeta_{e}$ will be set to zero throughout the remainder of this work.

**TABLE 1:** Interim analysis decision relative to observed outcomes at the end of the Phase II.

$\mathbb{P}\left(\theta_{\mathrm{TS}}>\zeta_{e}|D_{k}\right)>\tau_{S}$	$\mathbb{P}\left(\theta_{T}>\zeta_{e}|D_{k}\right)>\tau_{F}$	Population studied in Phase III
Yes	Yes	F&S
	No	S if $\mathbb{P}\left(\theta_{T}+\theta_{\mathrm{TS}}>\zeta_{e}|D_{k}\right)>\tau_{1}$
		Futility if $\mathbb{P}\left(\theta_{T}+\theta_{\mathrm{TS}}>\zeta_{e}|D_{k}\right)\leq \tau_{1}$
No	Yes	F
	No	Futility

### 3.4 Stage 2‐Phase III

**FIGURE 2:**
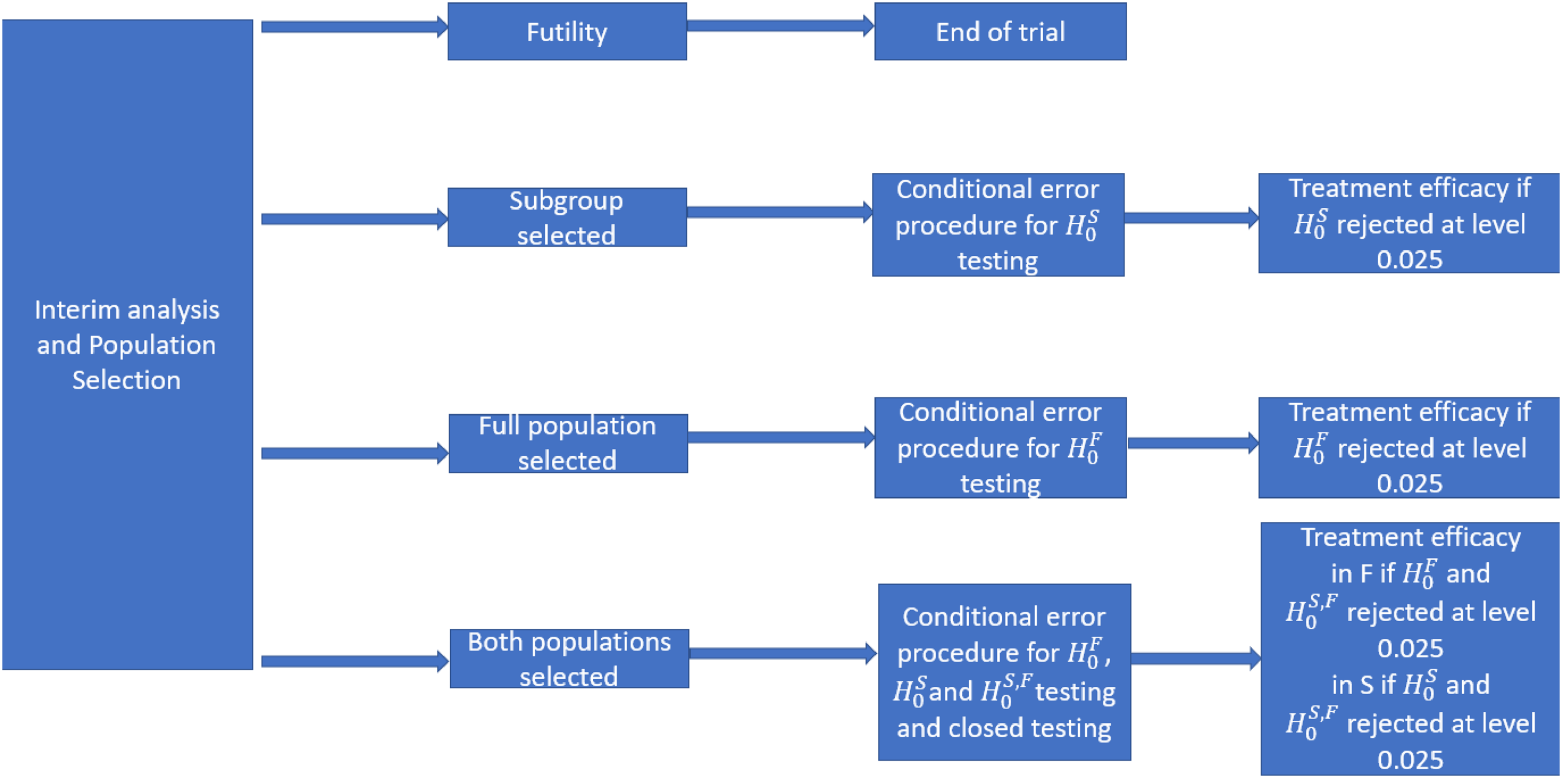
Representation of the trial tests at the end of the trial given the outcome of interim analysis.

If the trial progresses to the second stage, there are three possible scenarios: the trial may continue with F, proceed with S, or move forward with both F&S for the analysis, as illustrated in Figure 2. For the final analysis, a one‐sided Log Rank Test (LRT) is employed: $H_{0}:S_{0}\left(t\right)\geq S_{1}\left(t\right)$ versus $H_{1}:S_{0}\left(t\right)<S_{1}\left(t\right)$, with $S_{0}(t)$ and $S_{1}(t)$ representing the survival function of the control and treatment arm respectively, in the selected population. To account for the selection step and control the type I error rate, we use the conditional error function approach along with the Spiessens and Debois [11] testing method to address the correlation between the tests for F and S. If both population are selected to be studied in the final analysis, we also use a closed test procedure to ensure that the type I error rate is controlled at the desired level (0.025 one‐sided in our application) as explained in more detail in the following subsection.

#### 3.4.1 The Conditional Error Function Accounting for Correlation

The Conditional Error Function (CEF) approach utilized for the final analysis is proposed by Friede et al. [12] and has been applied in subsequent studies [18, 22]. This methodology is recognized for its effectiveness in controlling the type I error rate in adaptive designs, particularly when adjustments are made based on interim analysis outcomes. The use of the Spiessens and Debois [11] testing procedure enables the correlation between the tests for F and S to be accounted for. Indeed, this method takes into consideration the existing correlation between test statistics, denoted as $Z^{F}$ and $Z^{S}$, for F and S, respectively. The adoption of this approach supposes that for patients of the Phase II part, we have two distinct datasets: one consists of dichotomized survival data at time $t^{*}$, utilized during the interim analysis, and the other with full survival data, accessible only at the trial conclusion, which is used in the final analysis. Consequently, patients from the first stage within the selected group will continue their follow‐up until the final analysis is conducted.

Let $H_{0}^{F}$ represent the null hypothesis for F, $H_{0}^{S}$ denote the null hypothesis for S, and $H_{0}^{\left\{S,F\right\}}=H_{0}^{F}\cap H_{0}^{S}$ be the intersection null hypothesis. Let $Z_{1}^{F}$ and $Z_{1}^{S}$ be the observed normalized tests statistics for the Phase II time‐to‐event data in F and S, respectively. Similarly, let $Z_{1,b}^{F}$ and $Z_{1,b}^{S}$ be the tests statistics of Phase II binary data in F and S. It is also assumed that the correlation between $Z_{1}^{F}$ and $Z_{1,b}^{F}$ is ρ, that is, $\;\text{corr}\;\left(Z_{1}^{F},Z_{1,b}^{F}\right)=\rho$ (which is then reflected to the S statistics). Let $S_{1}^{F}=w_{1}Z_{1,b}^{F}$ (respectively, $S_{1,b}^{S}$ for the sub‐population) represent the weighted test statistics, where $w_{1}=\sqrt{n_{\text{phII}}/n_{\text{total}}}$ represents the ratio of the number of patients in Phase II to the total sample size, reflecting the proportion of data from Phase II in the overall analysis. For the Phase III data, let $Z_{2}^{F}$ (respectively, $Z_{2}^{S}$ for the sub‐population) be the test statistics. Define $S_{2}^{F}=w_{1}Z_{1}^{F}+w_{2}Z_{2}^{F}$ (respectively, $S_{2}^{S}$ for the sub‐population), where $w_{2}=\sqrt{1-w_{1}}$ (with $w_{1}^{2}+w_{2}^{2}=1$) represents the weight for the Phase III portion of the data relative to the total sample size. As described by Friede et al. [12] under $H_{0}^{\left\{S,F\right\}}$, the weighted test statistics are correlated and follow a Multivariate Normal distribution. In our specific situation, this can be represented as follows:

(1) $$\left(\begin{array}{c} S_{1}^{F}\\ S_{1}^{S}\\ S_{2}^{F}\\ S_{2}^{S} \end{array}\right)∼ℳN\left(\left(\begin{array}{c} 0\\ 0\\ 0\\ 0 \end{array}\right),\left(\begin{array}{cccc} w_{1}^{2} & w_{1}^{2}\sqrt{\tau } & \rho w_{1}^{2} & \rho w_{1}^{2}\sqrt{\tau }\\ w_{1}^{2}\sqrt{\tau } & w_{1}^{2} & \rho w_{1}^{2}\sqrt{\tau } & \rho w_{1}^{2}\\ \rho w_{1}^{2} & \rho w_{1}^{2}\sqrt{\tau } & 1 & \sqrt{\tau }\\ \rho w_{1}^{2}\sqrt{\tau } & \rho w_{1}^{2} & \sqrt{\tau } & 1 \end{array}\right)\right)$$

where τ represents the prevalence of the subgroup as in Friede et al. [12]. At interim analysis, early termination of the trial can be considered if the observed test statistic exceeds a fix threshold, similarly to group sequential designs. In our design, we do not consider early termination for efficacy at interim analysis, therefore the computation of the threshold value for the final analysis is solely dependent on the desired overall type I error rate. This threshold is calculated based on Equation (1) and $P\left(\;\max\;\left(S_{2}^{F},S_{2}^{S}\right)\geq c_{2}\right)=\alpha$. To be considered positive at the final analysis, the final statistical test must achieve at least a $c_{2}$ threshold, given $\left(s_{1}^{F},s_{1}^{S}\right)$ as detailed later. As described in Figure 2, at interim analysis, four decisions can be taken, either the trial stops for futility, or it continues only in F or only in S, or both population are analyzed at the end of the trial. The first stage data is introduced in the final test thanks to the described method.

If only one population is selected for the final analysis, the individual population null hypothesis, either $H_{0}^{F}$ for the full population or $H_{0}^{S}$ for the sub‐population, is rejected if its individual test statistic value is higher than the corrected critical value calculated as $Z_{2}^{F}>\frac{c_{2}-s_{1}^{F}\rho }{w_{2}}$ for the full population or $Z_{2}^{S}>\frac{c_{2}-s_{1}^{S}\rho }{w_{2}}$ for the sub‐population. These values are derived from the conditional error expressed as $P\left(S_{2}\geq c_{2}\;|\;s_{1},H_{0}\right)$, where we have omitted the population superscript for clarity.

If both population are selected for final analysis, then the intersection hypothesis also needs to be rejected. For the intersection hypothesis, the critical *p*‐value threshold $p_{\text{thresh}}$ is computed through the following equation, which takes into account the results from stage 1 tests $s_{1}^{F}$ and $s_{1}^{S}$: $p_{\text{thresh}}=1-P\left(Z_{2}^{F}<\frac{c_{2}-s_{1}^{F}\rho }{w_{2}},Z_{2}^{S}<\frac{c_{2}-s_{1}^{S}\rho }{w_{2}}\right)$. Letting $Z_{\max}=\max\;\left(Z_{2}^{F},Z_{2}^{S}\right)$ be the maximum observed test statistic, the intersection *p*‐value is $p^{S,F}=1-\int_{-\infty }^{Z_{\max}}\Phi \left(\frac{Z_{\max}-\sqrt{\tau }z}{\sqrt{1-\tau }}\right)\phi \left(z\right)\mathrm{dz}$. The intersection hypothesis is then rejected if $p^{S,F}<p_{\text{thresh}}$. Finally, a closed test procedure is considered for rejecting $H_{0}^{F}$ or $H_{0}^{S}$, meaning that individual and intersection hypothesis need to be rejected to conclude to a significant treatment effect in either of the populations.

#### 3.4.2 Frequentist Design

A frequentist trial is also conducted for comparison with Bayesian methods. We consider the method as presented by Friede et al. [18] in the ads *R* package. Similar to its Bayesian counterpart, selection is based on a threshold limit, utilizing statistical test values to select the population. To mimic the Bayesian method, S is selected if $Z_{1,b}^{S}>\tau_{S}^{*}$ and $Z_{1,b}^{F}<\tau_{F}^{*}$; F is chosen if $Z_{1,b}^{S}<\tau_{S}^{*}$ and $Z_{1,b}^{F}>\tau_{F}^{*}$; both F and S are subjected to testing if $Z_{1,b}^{S}>\tau_{S}^{*}$ and $Z_{1,b}^{F}>\tau_{F}^{*}$; and futility is declared at the interim analysis if none of these thresholds are met. The analysis employs the CEF method and combining statistical tests from both stages as described in detail above.

### 3.5 Prior Distributions Choice

**FIGURE 3:**
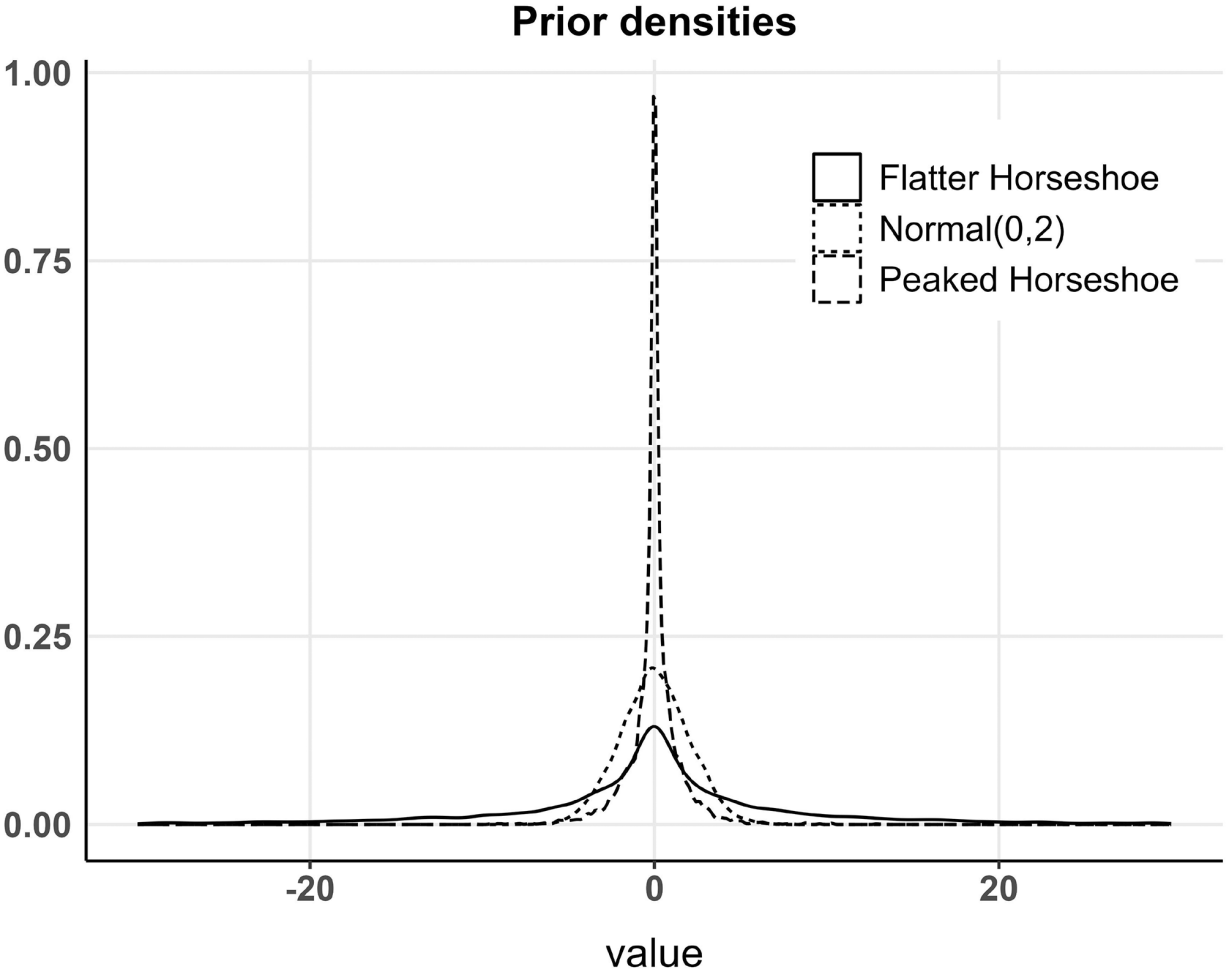
Prior distribution of regression parameters in the Bayesian design.

In this work, we aimed to evaluate the selection procedure using various prior distributions for $\theta_{S}$, $\theta_{T}$, and $\theta_{\mathrm{TS}}$. Initially, we selected two parametrizations of the horseshoe prior [23]. The horseshoe prior is known for its effectiveness in shrinking the posterior distributions of non‐influential covariates towards zero. For k={S,T,TS}, the horseshoe prior can be expressed as $\theta_{k}|\lambda_{k},\tau_{k}∼N\left(0,\tau_{k}^{2}\lambda_{k}^{2}\right)$, where $\lambda_{k}∼C^{+}\left(0,1\right)$, and $\;C^{+}\left(0,1\right)$ denotes a half‐Cauchy distribution. The parameter $\lambda_{k}$ is referred to as the local shrinkage parameter, and $\tau_{k}$ as the global shrinkage parameter. To complete the parametrization, a half t‐distribution was assigned to the global shrinkage parameter, denoted as $\tau_{k}∼T^{+}$. We compare two different parametrizations by varying the hyperparameters of the half‐t distribution. This approach yielded one prior distribution that gives more weight to zero, and another that is more spread out. The former is referred to as the “peaked horseshoe,” with $\tau_{k}∼T^{+}\left(0,1,1\right)$, indicating a tighter distribution around zero. The latter is called the “flatter horseshoe,” with $\tau_{k}∼T^{+}\left(0,10,1\right)$, indicating a broader, more dispersed distribution. We additionally explored another Bayesian prior, specifically a non‐informative normal distribution N(0,2) for the regression parameters $\theta_{k}$. Samples from the three prior densities are shown in Figure 3.

## 4 Simulation Study and Application to the Case Study

### 4.1 Simulation Setting

We assess the operating characteristics of the seamless hybrid design and its capability to accurately identify the population that benefits most from the treatment. Let $N_{F}$, $N_{S}$, and $N_{S^{C}}$ represent the sample sizes for the full population, the subgroup, and the complement of the subgroup ($S^{C}$), respectively. A total sample size of $N_{F}=190$ is allocated, with a prevalence of 0.7 for the subgroup S, resulting in $N_{S}=0.7\times 190=133$ and $N_{S^{C}}=57$. We present results for a subgroup S prevalence of 0.7, reflecting the case study trial, where the identified subgroup exhibited a prevalence around 0.6, and similar or higher prevalence values that were observed in other studies of interest (not described here). Consequently, our analysis focuses on high prevalence simulations to ensure that S maintains a larger sample size than $S^{C}$. However, results for a lower subgroup prevalence of 0.5 are available in the Supporting Information. Sample size computation was based on simulations involving various sample sizes for Phase II and Phase III. Regardless of ρ, which was fixed at a perfect correlation of 1 between the tests, scenarios 1 and 3 were used for hypothesis computation. Using the described design and algorithm for the frequentist method with $\tau_{F}^{*}=\tau_{S}^{*}=0$, we performed 1000 simulations for each case, exploring global sample sizes from 150 to 210 (in increments of 10), with 30 to 80 patients allocated to the first part. The sample size that controlled the Type I error rate at 0.05 (two‐sided) and achieved a power of approximately 0.8 was selected, assuming a median survival of 7 months in the control group and a hazard ratio of 0.7 under the alternative hypothesis using Log‐rank testing. For simplicity, we maintained a prevalence of 0.7 across all simulated samples.

We employ sample sizes of $N_{\mathrm{II}}=50$ for the second phase and $N_{\mathrm{III}}=140$ for the third phase of the study. Results for scenarios where an interim analysis is planned after 80 patients are given in the Supporting Information.

**FIGURE 4:**
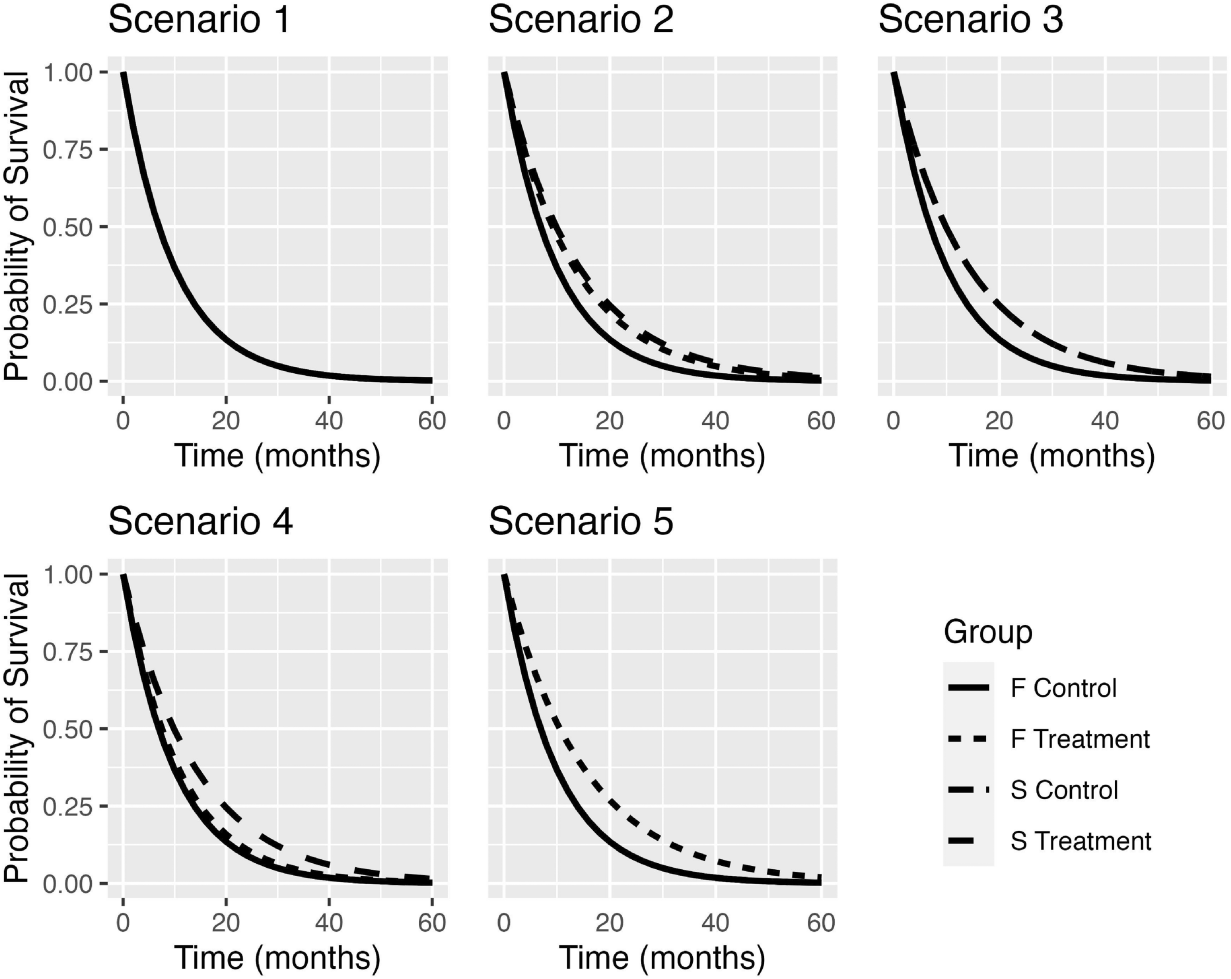
Survival times distribution for F and S populations in the five scenarios.

For patient simulation, hazard ratios were defined for both populations within the treatment group in comparison to the control in five scenarios. Table 2 provides a detailed description of the scenario parameters, whereas Figure 4 shows the resulting survival time distributions. Given that risks are proportional both within the S subgroup and its complement, risk proportionality between S and its complement is not anticipated in the control group. In our simulations, we did not encounter any issues with risk proportionality, as both populations S and its complement, $S^{C}$ were simulated to be proportional across treatment groups, with proportionality also observed approximately hold in the full population through their combination. However, in practice, this could pose greater challenges, as other covariates may influence survival, and the risk proportionality of $S^{C}$ is usually not studied. While this assumption was straightforward in our simulations, it should be carefully verified in the context of a real clinical trial.

**TABLE 2:** Simulation scenarios details.

Scenario	HR(F)	HR(S)	PST	PFT	PSC	PFC	Preferred selection
1	1	1	30%	30%	30%	30%	Futility
2	0.76	0.7	39%	42%	30%	30%	F&S, S, F
3	0.7	0.7	42%	42%	30%	30%	F, S, F&S
4	1.05	0.7	29%	42%	30%	30%	S, F&S
5	0.75	1	39%	30%	30%	30%	F, F&S

*Note:* The table columns include the scenario number, the Hazard Ratio (HR) for the treatment versus control for the full population, and the HR for the subgroup. PST, PFT, PSC, and PFC represent the 1‐year survival rates in the Treatment Subgroup, Treatment Full Population, Control Subgroup, and Control Full Population, respectively. In the last column, the populations deemed acceptable for selection are displayed, with the preferred option highlighted in bold.

The first scenario, termed the “Futility” scenario, was designed to assess the design's capability to identify a study as futile when the treatment does not benefit the F nor S. In the second scenario, the subgroup S experiences an increased benefit from the treatment compared to its complement, with a HR for S of 0.7 and for F of 0.76. The third scenario posits that F exhibits the same treatment effect as S, with HR(F)=HR(S)=0.7. In the fourth scenario, the hazard ratio for the full population HR(F) is 1.05 and HR(S)=0.7, indicating a strong negative effect in the complementary subgroup and maintaining the same beneficial effect in S. Given the high prevalence of S, achieving a hazard ratio greater than one in F implies that patients in $S^{C}$ subgroup have a significantly high HR. In the final scenario, the S population does not benefit from the treatment, exhibiting a hazard ratio of HR(S)=1, whereas its complementary population does benefit, with a hazard ratio for the full population HR(F)=0.75. This scenario illustrates a situation where the population benefiting from the treatment was incorrectly pre‐identified.

## 5 Results

The results of the simulations for the various methods under the range of scenarios described above are detailed in Tables 3 and 4. For simplicity, we assumed that the parameter ρ is known and set at a value of 0.8. This value is based on the correlation estimated via simulations conducted at the trial planning stage. These simulations, conducted across all relevant scenarios, including the null hypothesis and alternative hypotheses representing the desired hazard ratios, estimated the correlation between the test values in the binary Phase II and survival outcomes in Phase III. These simulations yielded an average correlation value of approximately 0.8 across all scenarios in our setting.

**TABLE 3:** Results of the simulated scenarios with a prevalence of 0.7 for S, with $\tau_{F}=0.7$, $\tau_{S}=0.5$, and $\tau_{1}=0$.

Scenario	Model	Futility	F	S	S&F	Ftot	Stot	False positive rate
1	Peaked horseshoe	**48.1**	5.7 (9%)	40.1 (5%)	6.1 (8%)	1	2.5	3
	Flatter horseshoe	**45.5**	8.8 (9%)	39.8 (5%)	5.9 (5%)	1.1	2.3	3.1
	Normal (0.2)	**27**	26.9 (6%)	43.1 (5%)	3 (7%)	1.8	2.3	3.9
	Freq ($\tau_{F}^{*}=\tau_{S}^{*}=0$)	**44.3**	9 (4%)	8.3 (4%)	38.4 (4%)	1.9	1.8	2.2
	Freq ($\tau_{F}^{*}=0,\tau_{S}^{*}=0.5$)	**49.6**	20.2 (5%)	3 (3%)	27.2 (5%)	2.4	1.5	2.5
	Freq ($\tau_{F}^{*}=0.5,\tau_{S}^{*}=0$)	**50.5**	2.8 (4%)	18.3 (3%)	28.4 (5%)	1.5	1.9	2

Scenario	Model	Futility	F	S	S&F	Ftot	Stot	Overall power
2	Peaked horseshoe	12.5	6.6 (89%)	54.7 (80%)	**26.2 (83%)**	27.7	65.4	71.3
	Flatter horseshoe	11.8	9.9 (88%)	53.4 (80%)	**24.9 (84%)**	29.6	63.5	72.2
	Normal (0.2)	7.1	28.2 (83)	50.2 (80%)	**14.5 (88%)**	36.1	52.9	76.3
	Freq ($\tau_{F}^{*}=\tau_{S}^{*}=0$)	9.9	4.6 (72%)	4.7 (64%)	**80.8 (76%)**	64.7	64.4	67.7
	Freq ($\tau_{F}^{*}=0,\tau_{S}^{*}=0.5$)	13.4	13 (68%)	1.2 (67%)	**72.4 (78%)**	65.2	57.2	66
	Freq ($\tau_{F}^{*}=0.5,\tau_{S}^{*}=0$)	13.2	1.3 (77%)	15.9 (70%)	**69.6 (79%)**	56	66.2	67.2
3	Peaked horseshoe	14.2	**17.9 (94%)**	34.8 (78%)	33.1 (90%)	46.8	57	73.9
	Flatter horseshoe	12.5	**25.4 (93%)**	33.7 (79%)	28.4 (90%)	49.2	52.2	75.7
	Normal (0.2)	7.2	**43.5 (92%)**	31.5 (79%)	17.8 (93%)	56.6	41.5	81.6
	Freq ($\tau_{F}^{*}=\tau_{S}^{*}=0$)	7.5	**7 (89%)**	1.9 (68%)	83.6 (81%)	74.3	69.4	75.6
	Freq ($\tau_{F}^{*}=0,\tau_{S}^{*}=0.5$)	8.8	**17.6 (83%)**	0.6 (67%)	73 (84%)	76	61.8	76.4
	Freq ($\tau_{F}^{*}=0.5,\tau_{S}^{*}=0$)	11.4	**3.1 (90%)**	8.9 (64%)	76.6 (84%)	66.8	69.7	72.5
4	Peaked horseshoe	2.7	0 (0%)	**95.8 (79%)**	1.5 (33%)	0.5	76.4	76.4
	Flatter horseshoe	2.4	0.3 (33%)	**95.4 (79%)**	1.9 (42%)	0.9	76.2	76.3
	Normal (0.2)	2	0.8 (25%)	**95.6 (79%)**	1.6 (31%)	0.7	76.5	76.7
	Freq ($\tau_{F}^{*}=\tau_{S}^{*}=0$)	14.3	0.2 (0%)	**23.6 (74%)**	61.9 (26%)	16.4	33.8	33.8
	Freq ($\tau_{F}^{*}=0,\tau_{S}^{*}=0.5$)	24.3	2.1 (5%)	**13.6 (79%)**	60 (27%)	16.4	27.1	27.2
	Freq ($\tau_{F}^{*}=0.5,\tau_{S}^{*}=0$)	14.5	0 (0%)	**41.9 (77%)**	43.6 (31%)	13.7	46	46
5	Peaked horseshoe	2.6	**96.1 (100%)**	0.2 (0%)	1 (18%)	96.1	0.2	96.1
	Flatter horseshoe	1.7	**97.1 (100%)**	0.3 (33%)	0.9 (22%)	97.1	0.3	97.2
	Normal (0.2)	0.1	**99.4 (100%)**	0.2 (0%)	0.3 (67%)	99.3	0.2	99.3
	Freq ($\tau_{F}^{*}=\tau_{S}^{*}=0$)	4.5	**48.8 (100%)**	0 (0%)	46.7 (6%)	51.5	2.9	51.5
	Freq ($\tau_{F}^{*}=0,\tau_{S}^{*}=0.5$)	4.5	**65.3 (100%)**	0 (0%)	30.2 (8%)	67.5	2.4	67.5
	Freq ($\tau_{F}^{*}=0.5,\tau_{S}^{*}=0$)	11	**42.3 (100%)**	0.2 (0%)	46.5 (6%)	45.2	2.9	45.2

*Note:* The columns Futility, F, S, and S&F indicate the percentage of their selections at the interim analysis; the percentages of these selections that lead to positive outcomes at the final analysis are shown in parentheses. The results for the preferred populations are shown in bold, and for the acceptable ones, they are underlined. The Ftot and Stot columns present the proportions of studies where F and S were selected and found significant, respectively. “False Positive Rate” denotes the proportion of studies incorrectly identified as positive in the first scenario, whereas “Overall Power” represents the proportion of studies correctly identifying at least one target population with a statistically significant final test.

**TABLE 4:** Results of the simulated scenarios with a prevalence of 0.7 for S, with $\tau_{F}=0.7$, $\tau_{S}=0.5$, and $\tau_{1}=0.4$.

Scenario	Model	Futility	F	S	S&F	Ftot	Stot	False positive rate
1	Peaked horseshoe	**63.5**	6.3 (9%)	11.3 (6%)	18.9 (8%)	1	2.5	3
	Flatter horseshoe	**51.3**	8.8 (9%)	34 (6%)	5.9 (5%)	1.1	2.3	3.1
	Normal (0.2)	**38.6**	26.9 (6%)	31.5 (6%)	3 (7%)	1.8	2.2	3.8

Scenario	Model	Futility	F	S	S&F	Ftot	Stot	Overall power
2	Peaked horseshoe	14	6.6 (89%)	53.2 (81%)	**26.2 (83%)**	27.7	64.8	70.7
	Flatter horseshoe	13.9	9.9 (88%)	51.3 (81%)	**24.9 (84%)**	29.6	62.6	71.3
	Normal (0.2)	10.3	28.2 (83%)	47 (83%)	**14.5 (88%)**	36.1	51.8	75.2
3	Peaked horseshoe	14.4	**17.9 (94%)**	34.6 (78%)	33.1 (90%)	67.4	64.9	73.7
	Flatter horseshoe	12.8	**25.4 (93%)**	33.4 (79%)	28.4 (90%)	49.2	52	75.5
	Normal (0.2)	8.8	**43.5 (92%)**	29.9 (82%)	17.8 (93%)	56.6	41.1	81.2
4	Peaked horseshoe	9.9	0 (0%)	**88.6 (82%)**	1.5 (33%)	0.5	73.3	73.3
	Flatter horseshoe	10.5	0.3 (33%)	**87.3 (82%)**	1.9 (42%)	0.9	72.6	72.7
	Normal (0.2)	11.5	0.8 (25%)	**86.1 (83%)**	1.6 (31%)	0.7	71.8	72
5	Peaked horseshoe	2.6	**96.1 (99.9%)**	0.2 (0%)	1.1 (18%)	96.1	0.2	96.1
	Flatter horseshoe	1.7	**97.1 (100%)**	0.3 (33%)	0.9 (22%)	97.1	0.3	97.2
	Normal (0.2)	0.1	**99.4 (100%)**	0.2 (0%)	0.3 (67%)	99.3	0.2	99.3

*Note:* The columns Futility, F, S, and S&F indicate the percentage of their selections at the interim analysis; the percentages of these selections that lead to positive outcomes at the final analysis are shown in parentheses. The results for the preferred populations are shown in bold, and for the acceptable ones, they are underlined. The Ftot and Stot columns present the proportions of studies where F and S were selected and found significant, respectively. “False Positive Rate” denotes the proportion of studies incorrectly identified as positive in the first scenario, whereas “Overall Power” represents the proportion of studies correctly identifying at least one target population with a statistically significant final test.

In Table 3, we present findings for the methods where the futility threshold $\tau_{1}$ is established at 0, $\tau_{F}=0.7$ and $\tau_{S}=0.5$. Table 4 introduces a more stringent condition for the Bayesian methods we evaluated. Here, the $\tau_{1}$ threshold is adjusted to 0.4. For each scenario, the tables present the proportion of studies identified as futile at the interim analysis and the percentage of times each population is selected out of 1000 simulated trials. The columns labeled “Ftot” and “Stot” denote the proportion of studies where populations F and S were selected and showed significant results at the final analysis. Lastly, the “Overall power” is defined as the percentage of studies in which the final analysis detected a significant effect of the treatment in at least one of the right populations. The frequentist method is run with three distinct threshold configurations, namely ($\tau_{F}^{*}=0,\tau_{S}^{*}=0$), ($\tau_{F}^{*}=0,\tau_{S}^{*}=0.5$), and ($\tau_{F}^{*}=0.5,\tau_{S}^{*}=0$).

In Scenario 1, the control of the type I error rate, or the false positive rate, is effectively maintained by frequentist approaches. This could also result from the frequent declaration of studies as futile at the interim analysis. Moreover, when studies continue past this point, both populations F&S are often selected for further investigation, leading to a stricter testing procedure at the final analysis. Conversely, Bayesian methods exhibit a slight increase in the type I error rate, with an inflation of up to 1.5% compared to their frequentist counterparts. For Scenario 2, frequentist methods exhibit approximately 5% lower overall power compared to the Bayesian horseshoe prior model. Both types of methods are surpassed by the Bayesian Normal model, which is less stringent regarding futility at the interim analysis and selects more often than the others F for Phase III. In Scenario 3, frequentist methods tend to exhibit comparable power to the horseshoe models. Consistent with previous findings, the Normal prior Bayesian model emerges as the most powerful (at the cost of the greatest type I error rate inflation). In the last two scenarios, where the treatment efficacy is simulated in only one population, we observe a significant difference between Bayesian and frequentist methods. For instance, in the fourth scenario, there is a notable decrease in power across all frequentist methods. However, when a threshold is applied to population F, the power increases. Bayesian methods consistently select the correct population with high accuracy and perform equivalently to one another. Conversely, in Scenario 5, the frequentist model with a threshold applied to population S outperforms the other frequentist approaches. Bayesian methods almost always successfully reject the null hypothesis by selecting F.

From Table 4, it is evident that implementing a more stringent futility rule by setting the threshold τ=0.4 increases the proportion of studies classified as futile. Although this adjustment has a minimal impact on most scenarios, it is noteworthy that in Scenario 4, where the effect is pronounced in population S, the peaked horseshoe prior model outperforms others. This superiority arises because the probability $P\left(\theta_{T}>0\right)$ is less likely to meet the threshold due to the posterior concentrating around zero. Conversely, for $P\left(\theta_{\mathrm{TS}}>0\right)$ and $P\left(\theta_{T}+\theta_{\mathrm{TS}}>0\right)$, the threshold is slightly more often met, resulting in increased selection of S and, consequently, a marginal boost in power. In Scenario 3 and 5, the introduction of $\tau_{1}$ does not significantly alter outcomes, as the treatment demonstrates efficacy in population F in both scenarios.

Further analyses with ρ equal to 0.5 are shown in the Supporting Information to assess the impact of the correlation. Lowering the correlation factor of the two tests to 0.5 impacts the type one error rate and the power in most scenarios. As ρ is decreasing, the final threshold value to be reached is decreasing too. This increases the probability of a final significant test. It results on a higher type one error rate with respect using a higher ρ. This also leads to a major power increase for all scenarios. Only scenario 5 has limited impact as most of the studies are already positive. Such a low correlation is nevertheless unlikely in the context of this paper since the test statistic based on the survival time is strongly correlated to the survival rate at a particular timepoint.

Furthermore, the impact of the selection in the estimation was assessed in all 5 scenarios by differentiating the mean estimated hazard ratio to the hazard ratio used for simulation. It results in a limited bias in the final testing estimation which is equivalent (to two decimal places) across all selection models. Estimation bias is reported in Table 5 and is higher in S than F. It also appears that the more the selection is balanced between S and F, the more bias is observed on the estimate hazard ratio. For Scenario 1 and 5, there is almost no bias, while the scenarios with subgroup selection leads to an underestimation of the treatment effect with an estimated HR higher for scenarios 2–4. To correct underestimation in those scenarios, unbiased methods can also be used for the estimators such as the ones described in [24, 25].

**TABLE 5:** Estimated bias on the final logrank testing for both population tests around all scenarios from 1000 simulated trials.

Scenario	Bias F	Bias S
1	0.01	0.02
2	0.16	0.15
3	0.16	0.15
4	−0.06	0.15
5	−0.007	−0.03

### 5.1 Application to the Atalante‐1 Study Data

In this subsection, we briefly show the application of Bayesian and frequentist methods to the Atalante‐1 study data. The original trial did not incorporate selection strategies at the interim analysis, consequently continuing the study with the entire population. Applying the Bayesian and frequentist methods to this trial data reveals distinct approaches between methodologies: Bayesian methods preferentially select the S population at the interim analysis, whereas frequentist designs proceed with both F and S populations. The posterior probabilities of being greater than zero for $\theta_{T}$ and $\theta_{\mathrm{TS}}$ are detailed in Table 6. For frequentist approaches (grouped all together in the last line of Table 6), the interim analysis leads to the continuation with both F and S populations, driven by the $Z_{F}$ value reaching a sufficient threshold. Despite the different methodologies, all designs ultimately categorize the study as futile, with a final *p*‐value of 0.18 observed in S. This outcome was anticipated, given that the original trial design did not account for these methodological strategies, leading to a sample size and randomization scheme that were not optimized.

**TABLE 6:** Results obtained using the Atalante‐1 dataset.

Method	$\mathbb{P}\left(\theta_{T}>0\right)$	$\mathbb{P}\left(\theta_{\mathrm{TS}}>0\right)$	$Z_{1,b}^{F}$	$Z_{1,b}^{S}$	*p* 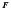	*p* 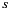	*p* 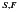
Peaked horseshoe	0.49	0.51	0.893	1.01	NA	0.18	NA
Flatter horseshoe	0.49	0.51	0.893	1.01	NA	0.18	NA
Normal (0.2)	0.58	0.74	0.893	1.01	NA	0.18	NA
Frequentist	NA	NA	0.893	1.01	0.4	0.18	0.24

## 6 Discussion

In this paper, we have proposed the use of a Bayesian approach to the selection of a population at an interim analysis within the framework of a seamless Phase II/III clinical trial. We have evaluated the operating characteristics of three Bayesian models, comparing these with a frequentist approach, each employing various thresholds.

From the results, it was observed that among the Bayesian models, those utilizing the horseshoe prior were the most inclined to declare studies futile at the interim analysis stage. The horseshoe prior is recognized for its effectiveness in variable selection, especially in contexts with a large number of covariates, by selecting a small number of significant variables and shrinking the others towards zero. However, in the specific context of selecting sub‐populations, the horseshoe prior may not be as advantageous due to its tendency to be overly conservative in declaring futility at interim analyses. In such scenarios, simpler priors, such as the normal prior distribution, might be preferable. The satisfactory performance of the normal prior distribution is also attributed to its reduced computational demand and enhanced convergence of Markov Chain Monte Carlo (MCMC) simulations. Although methods like Bayesian Model Averaging were considered, they were ultimately not included, as initial results did not demonstrate a significant advantage. On the other hand, the use of a normal prior distribution can increase the type I error rate, as it may lack conservatism during interim analysis, especially when only a few studies are stopped early. In scenarios evaluating the power, a normal prior tends to favor the selection of the F population over the F&S population, unlike the horseshoe prior. This leads to higher power, when F is one of the right population to be selected, because the final test is based solely on F tests, making it less conservative.

Simulation studies have also shown that in certain situations, using the frequentist statistical test value to choose between the full population and a subgroup can mistakenly lead to the decision to continue analyzing both populations. This error is particularly likely when a strong treatment effect is observed in the subgroup, artificially inflating the $Z^{F}$ value. For instance, in scenarios 4 and 5, where only one of the studied populations benefits from the treatment, a significant difference in overall power between the two types of methods is observed. In scenario 4, the $Z_{1,b}^{F}$ value reaches the $\tau_{F}^{*}$ threshold because S⊂F, implying an analysis in a non‐responding population and necessitating a stricter rule for the final analysis (as tests need to be performed in both populations). In scenario 5, the opposite effect occurs: with the HR=1 for the S subgroup, a $\tau_{S}^{*}$ threshold of zero is often reached, leading to the selection of both populations and, for the same reasons as in scenario 4, a loss of power. The use of logistic regression can overcome this issue. If a significant effect is observed in the S subgroup within the treatment group, the coefficient associated with the interaction term will deviate significantly from zero, whereas the coefficient for the full population will remain close to zero. The correlation between the tests of the two steps of the trial need to be assessed before running the trial. This correlation term can impact the final results as the final threshold can be lowered by an inappropriately chosen term and have a strong impact on the type one error rate. The use of a low assumed correlation in the Supporting Information only serves as a warning illustration since both tests should be highly correlated in order to have a well‐planned clinical trial.

During the planning stage of a real clinical study, the sample size should be carefully selected by evaluating various parameter combinations of ρ, τ and the censoring rate. The chosen sample size should achieve the desired statistical power on average. Finally, since simulation data was used to estimate the correlation between the binary and time‐to‐event tests, we did not overcome with the issue of having to estimate ρ from real clinical trial patient data. However, in practice it might be possible to estimate the value of the correlation parameter between the statistical tests using bootstrap sample of time‐to‐event data.

The difference between the Bayesian and frequentist methods lies in their selection rules. By applying certain mathematical transformations, it is conceivable to align Bayesian probability thresholds with their frequentist counterparts on the Z scale. However, even if a perfect correspondence between Bayesian and frequentist thresholds can be achieved, Bayesian methods introduce an additional layer of selection through the use of $\tau_{1}$. This additional criterion may explain why frequentist methods tend to select the F&S populations more frequently than the S population alone.

Other potential designs include proceeding with $S^{C}$ if a null effect is found in S. However, in our context, since the subgroup is identified before the trial, we did not explore this option. Sample size re‐estimation is another common adaptation in such contexts. We did not investigate the optimal sample size, as computations for all methods are time‐consuming, and substantially more simulations would be required. Nonetheless, as indicated in the Supporting Information, the larger the sample size at the interim analysis, the more accurate the selection of the population. However, it does not significantly enhance power in scenarios where S is selected, because the final sample size for S remains unchanged compared to when interim analysis is conducted with a smaller sample size. In this case, the slight improvement observed is attributed to enhanced selection accuracy.

## Conflicts of Interest

The authors declare no conflicts of interest.

## Supporting information


**Data S1.** Supplementary Information.

## Data Availability

No new data were created in this study. Access to Atalante‐1 data may be available upon request to the trial sponsor.
