# Supplementary material for: A Seamless Hybrid Phase II/III Design With Bayesian Interim Subgroup Selection
Source: Stat Med. 2025 Jun 3;44(13-14):e70144. doi: 10.1002/sim.70144 (PMC12134527; doi:10.1002/sim.70144)
Supplement: Supplementary file 1 — Data S1. Supplementary Information. [file SIM-44-0-s001.pdf]

Supplementary Materials of "A Seamless hybrid Phase II/III design with Bayesian interim subgroup selection"

In this document, we present results from additional parameters we evaluated. In Table 1, we present the outcomes for a prevalence of the S sub-population of 0.5. This naturally leads to a decrease in power in Scenario 2, where the subgroup is intended to be selected, while enhancing both selection accuracy and power in Scenario 5. Situations in which the subgroup influences the selection process are less powerful because the sample size of the final analysis decreases as the proportion of S becomes less significant. In Table 2, the sample size remains consistent with that of the main paper (190); however, the interim analysis is scheduled for a later time, when data from 80 patients in each group are available. Although this approach poses a risk in the event of an ineffective experimental treatment, by potentially exposing a greater number of patients to it, it facilitates more accurate selection in all other scenarios due to the availability of more information. This results in a slight increase in power as the selection process becomes more precise. We observe a similar trend to the interim analysis at 50 patients when we reduce the prevalence of the subgroup to 0.5, as illustrated in Table 4.

Table 1: Results of the simulated scenarios with a prevalence of 0.5 for S, with $\tau_F = 0.7$, $\tau_S = 0.5$, and $\tau_1 = 0$. The total sample size is set at 190, with an interim analysis conducted after assessing the 1-year survival rate of 50 patients. The columns Futility, F, S, and $S\&F$ indicate the percentage of their selections at the interim analysis; the percentages of these selections that lead to positive outcomes at the final analysis are shown in parentheses. The results for the preferred populations are shown in bold, and for the acceptable ones, they are underlined. The Ftot and Stot columns present the proportions of studies where F and S were selected and found significant, respectively. 'False Positive Rate' denotes the proportion of studies incorrectly identified as positive in the first scenario, whereas 'Overall Power' represents the proportion of studies correctly identifying at least one target population with a statistically significant final test.

Scenario	Model	Futility	F	S	$S\&F$	Ftot	Stot	False Positive Rate
1	Peaked Horseshoe	**47**	6.4 (14%)	39.7 (9%)	6.9 (13%)	1.9	4.6	5.5
	Flatter Horseshoe	**43.9**	8.7 (14%)	41 (10%)	6.4 (12%)	2.1	4.7	5.9
	Normal(0,2)	**29.5**	20.6 (11%)	44 (9%)	5.9 (12%)	3	4.5	6.8
	Freq($\tau_F^* = \tau_S^* = 0$)	**41.5**	12.6 (8%)	11.4 (11%)	34.5 (6%)	3.4	3.4	4.4
	Freq($\tau_F^* = 0, \tau_S^* = 0.5$)	**48.7**	21.6 (7%)	4.2 (14%)	25.5 (9%)	4	2.8	4.4
	Freq($\tau_F^* = 0.5, \tau_S^* = 0$)	**47.5**	6.6 (6%)	20.6 (8%)	25.3 (7%)	2.5	3.5	3.9

Scenario	Model	Futility	F	S	$S\&F$	Ftot	Stot	Overall Power
2	Peaked Horseshoe	13.7	6.5 (85%)	54.3 (78%)	**25.5 (78%)**	25.9	62.5	67.9
	Flatter Horseshoe	13.3	9.5 (86%)	51.9 (78%)	**25.3 (78%)**	28.3	60.1	68.2
	Normal(0,2)	8.6	20.8 (77%)	50.1 (79%)	**20.5 (80%)**	32.6	55.9	71.8
	Freq($\tau_F^* = \tau_S^* = 0$)	11.8	6.9 (65%)	9.3 (58%)	**72 (67%)**	53.1	54.8	57.9
	Freq($\tau_F^* = 0, \tau_S^* = 0.5$)	16.9	16.8 (69%)	4.2 (71%)	**62.1 (69%)**	54.7	47.1	57.3
	Freq($\tau_F^* = 0.5, \tau_S^* = 0$)	15.5	3.2 (72%)	21.3 (67%)	**60 (71%)**	45.3	57.4	59
3	Peaked Horseshoe	16.8	**18.1 (98%)**	29.4 (78%)	35.7 (85%)	49.4	53.6	71.3
	Flatter Horseshoe	14.5	**23.1 (98%)**	28.9 (79%)	33.5 (86%)	52.4	51.7	74.2
	Normal(0,2)	6.7	**38.6 (95%)**	26.1 (80%)	28.6 (87%)	62.3	46	82.4
	Freq($\tau_F^* = \tau_S^* = 0$)	6.7	**12 (93%)**	3.7 (62%)	77.6 (78%)	74	62.9	74
	Freq($\tau_F^* = 0, \tau_S^* = 0.5$)	8.6	**25.1 (91%)**	1.8 (78%)	64.5 (81%)	77	53.8	76.7
	Freq($\tau_F^* = 0.5, \tau_S^* = 0$)	11.8	**6.9 (97%)**	10.5 (61%)	70.8 (80%)	65.8	63.2	69.9
4	Peaked Horseshoe	2	0	**97.4 (77%)**	0.6 (16%)	0.1	75	75
	Flatter Horseshoe	2.5	0.1 (0%)	**97 (77%)**	0.4 (0%)	0	74.7	74.2
	Normal(0,2)	0.2	0.3 (0%)	**96.8 (77%)**	0.9 (33%)	0.3	74.5	74.5
	Freq($\tau_F^* = \tau_S^* = 0$)	11.4	0.1 (0%)	**58.1 (74%)**	30.4 (5%)	1.5	37.6	36.5
	Freq($\tau_F^* = 0, \tau_S^* = 0.5$)	32	1.7 (6%)	**34.3 (78%)**	32 (4%)	1.5	29.5	28.4
	Freq($\tau_F^* = 0.5, \tau_S^* = 0$)	18.7	0	**62 (76%)**	19.3 (5%)	1	49.1	48
5	Peaked Horseshoe	0	**99.6 (100%)**	0	0.4 (0%)	100	0	99.6
	Flatter Horseshoe	0.1	**99.5 (100%)**	0	0.4 (0%)	99.5	0	99.5
	Normal(0,2)	0	**99.5 (100%)**	0	0.5 (20%)	100	0.1	99.6
	Freq($\tau_F^* = \tau_S^* = 0$)	0	**52.7 (100%)**	0	47.3 (12%)	62	5.3	58.4
	Freq($\tau_F^* = 0, \tau_S^* = 0.5$)	0.1	**70.2 (100%)**	0	29.7 (15%)	76.9	4.6	74.8
	Freq($\tau_F^* = 0.5, \tau_S^* = 0$)	0.9	**53.2 (100%)**	0	45.9 (12%)	61.2	5.3	58.5

Table 2: Results of the simulated scenarios with a prevalence of 0.7 for S, with $\tau_F = 0.7$, $\tau_S = 0.5$, and $\tau_1 = 0$. The total sample size is set at 190, with an interim analysis conducted after assessing the 1-year survival rate of 80 patients. The columns Futility, F, S, and $S\&F$ indicate the percentage of their selections at the interim analysis; the percentages of these selections that lead to positive outcomes at the final analysis are shown in parentheses. The results for the preferred populations are shown in bold, and for the acceptable ones, they are underlined. The Ftot and Stot columns present the proportions of studies where F and S were selected and found significant, respectively. 'False Positive Rate' denotes the proportion of studies incorrectly identified as positive in the first scenario, whereas 'Overall Power' represents the proportion of studies correctly identifying at least one target population with a statistically significant final test.

Scenario	Model	Futility	F	S	$S\&F$	Ftot	Stot	False Positive Rate
1	Flatter Horseshoe	**43.4**	7.3 (15%)	43 (3%)	6.3 (13%)	2	2.1	3.4
	Peaked Horseshoe	**30.7**	21.3 (9%)	44.6 (4%)	3.4 (6%)	2.2	2	3.7
	Normal(0,2)	**25.5**	26.4 (8%)	45.3 (4%)	2.8 (4%)	2.3	1.9	3.7
	Freq($\tau_F = \tau_S = 0$)	**41.5**	10.3 (2%)	9.3 (3%)	38.9 (5%)	.4	1.8	2.5
	Freq($\tau_F = 0, \tau_S = 0.5$)	**48**	22.7 (4%)	2.8 (7%)	26.5 (7%)	2.5	2	2.9
	Freq($\tau_F = 0.5, \tau_S = 0$)	**49.9**	1.9 (5%)	21.4 (5%)	26.8 (7%)	1.7	2.9	3

Scenario	Model	Futility	F	S	$S\&F$	Ftot	Stot	Overall Power
2	Flatter Horseshoe	7.6	7.9 (87%)	53.2 (83%)	**31.3 (89%)**	34.7	72.3	79.2
	Peaked Horseshoe	4.9	23.4 (82%)	53.8 (84%)	**17.9 (91%)**	35.5	61.7	80.8
	Normal(0,2)	3.4	26.5 (81%)	52 (84%)	**18.1 (92%)**	38.1	60.3	81.8
	Freq($\tau_F = \tau_S = 0$)	6.3	3 (50%)	3.6 (56%)	**87.1 (78%)**	71.2	70	71.1
	Freq($\tau_F = 0, \tau_S = 0.5$)	8.5	9.4 (61%)	1.4 (79%)	**80.7 (80%)**	70.3	66.5	71.4
	Freq($\tau_F = 0.5, \tau_S = 0$)	7.9	1.4 (43%)	10.6 (64%)	**80.1 (80%)**	64.7	71.7	71.8
3	Flatter Horseshoe	7.9	**22.9 (94%)**	28.5 (85%)	40.7 (93%)	59.4	62.1	83.7
	Peaked Horseshoe	4.5	**42.2 (94%)**	30.3 (86%)	23 (95%)	61.5	47.9	87.6
	Normal(0,2)	3.3	**44.9 (93%)**	29 (86%)	22.8 (96%)	63.6	46.8	88.6
	Freq($\tau_F = \tau_S = 0$)	4	**5.3 (68%)**	1.2 (50%)	89.5 (82%)	77	74.9	78
	Freq($\tau_F = 0, \tau_S = 0.5$)	4.9	**13 (72%)**	3 (100%)	81.8 (85%)	79.7	72.5	79.6
	Freq($\tau_F = 0.5, \tau_S = 0$)	6.3	**3 (77%)**	5.6 (57%)	85.1 (84%)	73.8	74.7	77.1
4	Flatter Horseshoe	0.8	0	**98.4 (80%)**	0.8 (63%)	0.5	79.4	79.2
	Peaked Horseshoe	0.7	0.5 (20%)	**98.1 (81%)**	0.7 (71%)	0.6	80	80
	Normal(0,2)	0.3	0.3 (0%)	**98.7 (81%)**	0.7 (86%)	0.6	80.6	80.4
	Freq($\tau_F = \tau_S = 0$)	9.3	0	**22.1 (67%)**	68.6 (75%)	51.5	66.9	34.7
	Freq($\tau_F = 0, \tau_S = 0.5$)	16.9	1 (0%)	**14.5 (75%)**	67.6 (29%)	19.6	37.9	30.8
	Freq($\tau_F = 0.5, \tau_S = 0$)	9.3	0	**44.7 (75%)**	46 (38%)	17.5	51.9	50.9
5	Flatter Horseshoe	0.3	**99.7 (100%)**	0	0	99.7	0	99.7
	Peaked Horseshoe	1	**99 (100%)**	0	0	99	0	99
	Normal(0,2)	0.1	**99.9 (100%)**	0	0	99.9	0	99.9
	Freq($\tau_F = \tau_S = 0$)	2.1	**49.7 (99.5%)**	0	48.2 (10%)	97.9	4.8	54.3
	Freq($\tau_F = 0, \tau_S = 0.5$)	2.1	**68.6 (99.7%)**	0	29.3 (15%)	97.9	4.4	72.9
	Freq($\tau_F = 0.5, \tau_S = 0$)	5.3	**46.5 (100%)**	0	48.2 (10%)	94.7	4.8	51.3

Table 3: Results of the simulated scenarios with a prevalence of 0.5 for S, with $\tau_F = 0.7$, $\tau_S = 0.5$, and $\tau_1 = 0$. The total sample size is set at 190, with an interim analysis conducted after assessing the 1-year survival rate of 80 patients. The columns Futility, F, S, and $S\&F$ indicate the percentage of their selections at the interim analysis; the percentages of these selections that lead to positive outcomes at the final analysis are shown in parentheses. The results for the preferred populations are shown in bold, and for the acceptable ones, they are underlined. The Ftot and Stot columns present the proportions of studies where F and S were selected and found significant, respectively. 'False Positive Rate' denotes the proportion of studies incorrectly identified as positive in the first scenario, whereas 'Overall Power' represents the proportion of studies correctly identifying at least one target population with a statistically significant final test.

Scenario	Model	Futility	F	S	$S\&F$	Ftot	Stot	False Positive Rate
1	Flatter Horseshoe	**42.4**	7.7 (14%)	43.3 (5%)	6.6 (9%)	2	2.8	3.9
	Peaked Horseshoe	**31.2**	18.3 (11%)	45.4 (4%)	5.1 (10%)	1.9	2.4	4.3
	Normal(0,2)	**27.7**	22 (10%)	45 (4%)	5.3 (9%)	2.7	2.3	4.6
	Freq($\tau_F = \tau_S = 0$)	**40.9**	11.8 (2%)	12.6 (5%)	34.7 (5%)	2	2.7	2.7
	Freq($\tau_F = 0, \tau_S = 0.5$)	**48**	20.6 (5%)	5.5 (4%)	25.9 (6%)	2.6	1.8	2.9
	Freq($\tau_F = 0.5, \tau_S = 0$)	**48.7**	4 (5%)	22.5 (6%)	24.8 (6%)	1.7	2.8	3.2

Scenario	Model	Futility	F	S	$S\&F$	Ftot	Stot	Overall Power
2	Flatter Horseshoe	8.3	7.1 (83%)	55.2 (71%)	**29.4 (83%)**	30.3	63.6	69.7
	Peaked Horseshoe	6.3	17.3 (82%)	54.1 (72%)	**22.3 (81%)**	32.2	57.2	71.2
	Normal(0,2)	5.6	20.5 (80%)	50.8 (71%)	**23.1 (81%)**	35.1	54.8	71.6
	Freq($\tau_F = \tau_S = 0$)	6.1	5.4 (41%)	6.3 (63%)	**82.2 (65%)**	55.1	58.2	59.8
	Freq($\tau_F = 0, \tau_S = 0.5$)	9	14.5 (54%)	3.4 (85%)	**73.1 (68%)**	59	52.6	60.6
	Freq($\tau_F = 0.5, \tau_S = 0$)	8.7	2.8 (61%)	18.3 (66%)	**70.2 (69%)**	50.4	61.2	62.3
3	Flatter Horseshoe	8.8	**27.1 (97%)**	21.6 (81%)	42.5 (87%)	64.1	54.5	80.9
	Peaked Horseshoe	4.2	**41.5 (96%)**	21.3 (84%)	33 (88%)	69.5	46.9	87.1
	Normal(0,2)	2.7	**45.6 (96%)**	19.5 (83%)	32.2 (90%)	73.4	45.2	89
	Freq($\tau_F = \tau_S = 0$)	3.2	**8.3 (87%)**	1.5 (67%)	87 (76%)	75	67.1	74.3
	Freq($\tau_F = 0, \tau_S = 0.5$)	4	**19.5 (90%)**	0.7 (86%)	75.8 (80%)	78.2	63.4	78.5
	Freq($\tau_F = 0.5, \tau_S = 0$)	5.6	**5.9 (90%)**	5.1 (71%)	83.4 (77%)	72	67.8	72.8
4	Flatter Horseshoe	0.6	0	**99.2 (78%)**	0.2 (50%)	0.1	77.5	77.5
	Peaked Horseshoe	0.5	0.1 (0%)	**99.1 (78%)**	0.3 (33%)	0.1	77.5	77.4
	Normal(0,2)	0.2	0.1 (0%)	**99.3 (78%)**	0.4 (25%)	0.1	77.8	77.5
	Freq($\tau_F = \tau_S = 0$)	11.4	0.1 (0%)	**58.1 (74%)**	30.4 (5%)	1.5	58.1	44.3
	Freq($\tau_F = 0, \tau_S = 0.5$)	23.1	0.4 (0%)	**46.4 (79%)**	30.1 (5%)	1.5	51.7	38
	Freq($\tau_F = 0.5, \tau_S = 0$)	11.5	0	**71.7 (76%)**	16.8 (7%)	1.2	66.2	55.9
5	Flatter Horseshoe	0.2	**99.8 (100%)**	0	0	99.8	0	99.8
	Peaked Horseshoe	0.7	**99.3 (100%)**	0	0	99.3	0	99.3
	Normal(0,2)	0	**100 (100%)**	0	0	100	0	100
	Freq($\tau_F = \tau_S = 0$)	0	**52.7 (100%)**	0	47.3 (12%)	100	5.6	58.4
	Freq($\tau_F = 0, \tau_S = 0.5$)	0	**68.6 (100%)**	0	31.4 (15%)	100	4.7	73.4
	Freq($\tau_F = 0.5, \tau_S = 0$)	0.1	**52.6 (100%)**	0	47.3 (12%)	99.9	5.6	58.3

Table 4: Results of the simulated scenarios with a prevalence of 0.7 for S, with $\tau_F = 0.7$, $\tau_S = 0.5$, and $\tau_1 = 0$. The total sample size is set at 190, with an interim analysis conducted after assessing the 1-year survival rate of 50 patients. Correlation between binary and survival testing is set to $\rho = 0.5$ The columns Futility, F, S, and $S\&F$ indicate the percentage of their selections at the interim analysis; the percentages of these selections that lead to positive outcomes at the final analysis are shown in parentheses. The results for the preferred populations are shown in bold, and for the acceptable ones, they are underlined. The Ftot and Stot columns present the proportions of studies where F and S were selected and found significant, respectively. 'False Positive Rate' denotes the proportion of studies incorrectly identified as positive in the first scenario, whereas 'Overall Power' represents the proportion of studies correctly identifying at least one target population with a statistically significant final test.

Scenario	Model	Futility	F	S	$S\&F$	Ftot	Stot	False Positive Rate
1	Flatter Horseshoe	**45.5**	8.8(14%)	39.8(10%)	5.9 (10%)	1.8	4.6	5.8
	Peaked Horseshoe	**48.1**	5.7 (14%)	40.1(10%)	6.1 (10%)	1.9	4.6	5.4
	Normal(0,2)	**27**	26.9 (13%)	43.1 (11%)	3 (10%)	5	3.7	8.4

Scenario	Model	Futility	F	S	$S\&F$	Ftot	Stot	Overall Power
2	Flatter Horseshoe	11.8	9.9 (95%)	53.4 (92%)	**24.9 (95%)**	33.1	72.8	82.2
	Peaked Horseshoe	12.5	6.6 (95%)	54.7 (92%)	**26.2 (95%)**	31.2	75.2	81.5
	Normal(0,2)	7.1	28.2(95%)	50.2 (92%)	**14.5 (95%)**	40.3	60.4	86.9
3	Flatter Horseshoe	12.5	**25.4 (99%)**	33.7 (92%)	28.4 (96%)	52.5	58.3	83.4
	Peaked Horseshoe	14.2	**17.9 (99%)**	34.8 (92%)	33.1 (96%)	49.5	63.8	81.5
	Normal(0,2)	7.2	**43.5 (99%)**	31.5 (92%)	17.8 (96%)	60.6	46.2	89.4
4	Flatter Horseshoe	2.4	0.3 (33%)	**95.4 (93%)**	1.9 (66%)	1.3	90.1	90.1
	Peaked Horseshoe	2.7	0 (0%)	**95.8 (93%)**	1.5 (66%)	0.1	90.1	90.1
	Normal(0,2)	2	0.8 (42%)	**95.6 (93%)**	1.6 (71%)	1.5	90	90.3
5	Flatter Horseshoe	1.7	**97.1(100%)**	0.3(33%)	0.9(33%)	97.1	0.4	97.5
	Peaked Horseshoe	2.6	**96.1(100%)**	0.2(50%)	1(30%)	96.1	0.4	96.5
	Normal(0,2)	0.1	**99.4(100%)**	0.2(50%)	0.3(33%)	99.3	0	99.5
